# Medical “Etiquette”

**Published:** 1893-05

**Authors:** 


### MEDICAL "ETIQUETTE."

Within the present week in this city occurred an incident that is typical of the extreme to which professional etiquette may be carried. A patient lay ill of a raging fever, whose progress, apparently, had not been prevented or mitigated by the remedies prescribed by the doctor "in charge" of the case. That doctor was not entirely satisfactory to the patient, and was even distasteful to the latter, but had been called in merely because he happened to be near at hand. The sick man protested strongly against the continuance of the seemingly useless treatment, and insisted that the family physician should be called. In a spirit of courtesy, the attending physician was told of the facts and of the wish of the patient. The family doctor was summoned, and was met by the attending physician. The two doctors had a private consultation, and as a result the attending physician announced that he would "remain in charge," despite the objections of the patient and the family, despite the knowledge that his every visit so irritated and exasperated the patient as to increase the fever of the helpless, but thoroughly indignant victim. The family then made an almost piteous appeal to the family physician to visit the patient, whose condition was such as to occasion grave alarm and the most depressing fears; but the reply was made that no reputable physician in Massachusetts would so imperil his standing under the rules which governed physicians as to attend a patient so long as the attending physician refused his consent.

The facts in the case are indisputable, and are given from personal knowledge. The consideration that under the circumstances the very life of the patient might be endangered, made no impression upon the two doctors, who looked only to the "etiquette" of their profession. If the medical practitioners stated the rule correctly, one doctor in this commonwealth may have the conceded right to prohibit the attendance of another doctor on a patient, no matter what the wishes or state of the sick man may be, and as a result of an exaggerated deference to this "etiquette," it is possible to suppose that a patient, constantly irritated and excited under a condition that demands rest and quiet, may die, but the senseless etiquette will have been observed.

